# Biophysical characterization of the inactivation of *E. coli* transketolase by aqueous co-solvents

**DOI:** 10.1038/s41598-021-03001-8

**Published:** 2021-12-08

**Authors:** Phattaraporn Morris, Ribia García-Arrazola, Leonardo Rios-Solis, Paul A. Dalby

**Affiliations:** 1 Department of Biochemical Engineering, University College London, Bernard Katz Building, Gower Street, London, WC1E 6BT UK; 2 Chemical Metrology and Biometry Department, National Institute of Metrology, 3/4-5 Moo 3, Klong 5, Klong Luang, 12120 Pathumthani Thailand; 3 Institute for Bioengineering, School of Engineering, University of Edinburgh, Edinburgh, EH9 3JL UK; 4 Centre for Synthetic and Systems Biology (SynthSys), University of Edinburgh, King’s Buildings, Edinburgh, EH9 3JL UK

**Keywords:** Biophysics, Biotechnology

## Abstract

Transketolase (TK) has been previously engineered, using semi-rational directed evolution and substrate walking, to accept increasingly aliphatic, cyclic, and then aromatic substrates. This has ultimately led to the poor water solubility of new substrates, as a potential bottleneck to further exploitation of this enzyme in biocatalysis. Here we used a range of biophysical studies to characterise the response of both *E. coli* apo- and holo-TK activity and structure to a range of polar organic co-solvents: acetonitrile (AcCN), *n*-butanol (nBuOH), ethyl acetate (EtOAc), isopropanol (iPrOH), and tetrahydrofuran (THF). The mechanism of enzyme deactivation was found to be predominantly via solvent-induced local unfolding. Holo-TK is thermodynamically more stable than apo-TK and yet for four of the five co-solvents it retained less activity than apo-TK after exposure to organic solvents, indicating that solvent tolerance was not simply correlated to global conformational stability. The co-solvent concentrations required for complete enzyme inactivation was inversely proportional to co-solvent log(P), while the unfolding rate was directly proportional, indicating that the solvents interact with and partially unfold the enzyme through hydrophobic contacts. Small amounts of aggregate formed in some cases, but this was not sufficient to explain the enzyme inactivation. TK was found to be tolerant to 15% (v/v) iPrOH, 10% (v/v) AcCN, or 6% (v/v) nBuOH over 3 h. This work indicates that future attempts to engineer the enzyme to better tolerate co-solvents should focus on increasing the stability of the protein to local unfolding, particularly in and around the cofactor-binding loops.

## Introduction

Biocatalysis has become increasingly powerful for the efficient synthesis of optically pure pharmaceuticals, agrochemicals, food ingredients, nutraceuticals, and fragrances^1–8^. A key challenge in biocatalysis is to overcome the poor solubility in aqueous media for many organic molecules of interest^9,10^. Eutectic solvents have emerged as an interesting alternative in this topic^11,12^. While biocatalysts are often limited in terms of their stability under industrial process conditions, directed evolution and rational enzyme engineering can potentially be used to address many of these issues, which include poor stability at extremes of pH, temperature and under oxidative stress^13–16^.

Many syntheses involve organic substrates or products that are poorly soluble in water, or are sensitive to aqueous degradation, resulting in a need for enzymes that operate efficiently in organic media, aqueous-organic mixtures, or aqueous two-phase systems^17,18^. Many enzymes can function in both aqueous and near-anhydrous organic solvents^19,20^, and in some cases organic solvents are found to improve enzyme stability or even alter their enantioselectivity^21–23^. The impact of solvents on protein structure and dynamics^24^ have been characterised extensively in near-anhydrous systems where the enzymes are typically freeze-dried or chemically cross-linked prior to addition to organic solvents. However, most enzymes are rapidly inactivated even at low concentrations of organic co-solvents, and yet the mechanisms of activity loss remain unclear^25,26^. Activity loss is typically solvent concentration dependent, but varies with organic solvent, potentially depending on solvent hydrophobicity^27,28^.

Enzymes typically have lower reaction rates in organic media relative to those in aqueous solutions, and yet they are often found to retain their overall native structure^29^. However, direct structural investigations of enzymes that are either suspended or solubilized into organic media are difficult, and so in the past, activity assays have been relied upon to give an indirect probe of protein structure and function. As a result, the mechanisms leading to partial inactivation of enzymes in organic solvents are still not clear, or generalizable to a wide range of enzymes such that they elucidate the relative roles of protein-solvent interactions, surface or active-site dehydration, structural denaturation, partial unfolding, subunit or cofactor dissociation, and enzyme aggregation^30–32^.

To take advantage of the benefits of organic solvents in biocatalysis, many efforts have been made to enhance enzyme activity and stability in aqueous-organic mixtures, particularly using enzyme immobilization^33–35^, directed evolution^36,37^ and recently viral nanoparticle-encapsulated enzymes among other strategies^38^. These studies have provided useful insights into the impact of organic solvents on enzyme structure and activity. However, novel rational or computational design of organic solvent tolerant enzymes, and their directed evolution, could be significantly improved if the mechanisms by which organic solvents cause enzyme inactivation were better understood.

**Figure 1:**
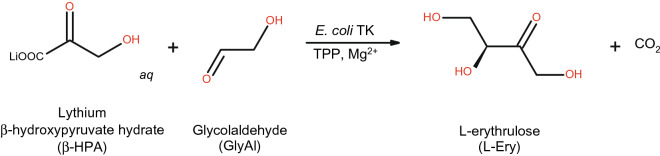
Reaction of transketolase.

*Escherichia coli* transketolase (TK) (EC 2.2.1.1) is a homodimeric enzyme in which each 78 kDa subunit comprises three domains, and the apo-TK homodimer binds two Ca^2+^ or Mg^2+^ ions, and two thiamine diphosphate (TPP) cofactors to form the active holo-TK enzyme^39,40^. It catalyses the stereoselective transfer of a two-carbon ketol group to an aldose sugar producing a new asymmetric carbon–carbon bond, at two steps within the reductive pentose phosphate pathway^41^. The reaction is rendered irreversible in vitro by the release of carbon dioxide, when using β-hydroxypyruvate as the ketol donor (Fig. 1). These features make the enzyme attractive for the biocatalytic synthesis of complex carbohydrates and their analogues^42–44^, as applied in the industrial synthesis of xylulose-5-phosphate^45,46^.

The synthetic potential of *E. coli* TK has already been significantly improved by successive rounds of directed evolution with smart libraries^47,48^, to enhance activity towards polar^49,50^, and increasingly aliphatic^51–53^, and heterocyclic substrates^51,54^, to enhance and reverse enantioselectivity^55^. Similar engineering of substrate specificity has been achieved in TK from other organisms^56,57^. De novo pathways containing improved TK variants have also been designed for the synthesis of optically pure high-value amino-diols^58,59^. Most recently the “substrate-walk” by directed evolution was continued to obtain variant "3 M" which introduced new activity towards substituted benzaldehyde substrates^60^, and then evolved further for simultaneous acceptance of pyruvate in the reaction with 3-formylbenzoic acid (3-FBA)^61^. However, many new benzaldehydes were too insoluble to test their activity, which has increased the need to explore the use of TK variants in the presence of organic co-solvents.

While we have previously characterised the activity, thermostability and aggregation of transketolase at a wide range of pH, temperature, and chemical denaturant concentrations^62,63^, its stability in a range of organic co-solvents has not been previously determined. TK unfolds irreversibly with urea, heat, or at low pH, and then aggregates, except in urea. Folded yet inactive states form during early urea-unfolding, and at high pH, in which the cofactors remain bound. The enzyme then partially unfolds at higher urea prior to dissociation of the monomers.

Several differences in the protein conformation of the holo and apo TK have been previously observed, for example the secondary and tertiary structure of holo-TK remained constant from pH 5 to 11, whereas the apo-TK secondary structure content increased with pH^62^. The effects of varying the Mg^2+^ and TPP cofactor concentrations on TK activity have been well characterised, showing inactivity in apo-TK and the concentrations required to effectively reach maximum activity in holo-TK^64^. Recently, a mutation to proline in one of the two TK cofactor-binding loops was found to stabilise TK against aggregation at elevated temperatures^14^. Further engineering of these flexible loops led to improved thermostability of the wild-type TK^48^, while their inclusion into variant 3 M, along with two stabilising mutations at residues that were dynamically coupled to the cofactor binding loops, resulted in variants 5 M and 7 M which had improved stability and activity towards the aromatic aldehydes^65^. Thus, one or more of several potential mechanisms could influence TK stability and activity in the presence of organic co-solvents, including: (i) global unfolding, (ii) aggregation; (iii) destabilisation of local structure or domains; (iv) cofactor dissociation; and v modulating the accessibility of water molecules or substrates into the active site.

Here we have characterised the impact of different co-solvents used in the pharmaceutical industry, including acetonitrile (AcCN), *n*-butanol (nBuOH), ethyl acetate (EtOAc), isopropanol (iPrOH), and tetrahydrofuran (THF), on both apo-TK and holo-TK activity and conformation, and correlated this to the physico-chemical properties of the solvents. We also determined the extent to which global and local protein unfolding, and aggregation play a role in solvent-induced enzyme inactivation. This work has provided useful insights that will guide the future engineering of TK and other similarly complex enzymes.

## Results

### Transketolase activity in organic co-solvents

Protein engineering of the cofactor-binding loops in TK was previously shown to improve the stability of TK to thermal aggregation^14,48,65^. They are also known to become more structured upon formation of holo-TK^40^, leading to increased thermostability compared to apo-TK^62,63,66^. Therefore, to understand the effect of polar organic co-solvents upon TK stability, including any potential role of the cofactor-binding loops, both apo-TK and holo-TK were incubated with five selected polar co-solvents at a range of concentrations from 0 to 30% (v/v). In the first case, apo-TK was incubated with the organic solvents for 3 h and then incubated with the cofactor to form the holo-TK before diluting and adding the substrates. In the second case, to test holo-TK, the apo-TK was first incubated with cofactors to form the holo-TK, and then incubated with organic solvents for 3 h before diluting and adding the substrates. In both cases, the solvent incubations were shaken at 1000 rpm to ensure that the least miscible solvents, ethyl acetate (EtOAc), and *n*-butanol (nBuOH) were either fully dissolved or formed into emulsions at the higher concentrations (biphasic systems were only formed for EtOAc and nBuOH at concentrations of 8.3 and 7.3% v/v respectively).

**Figure 2:**
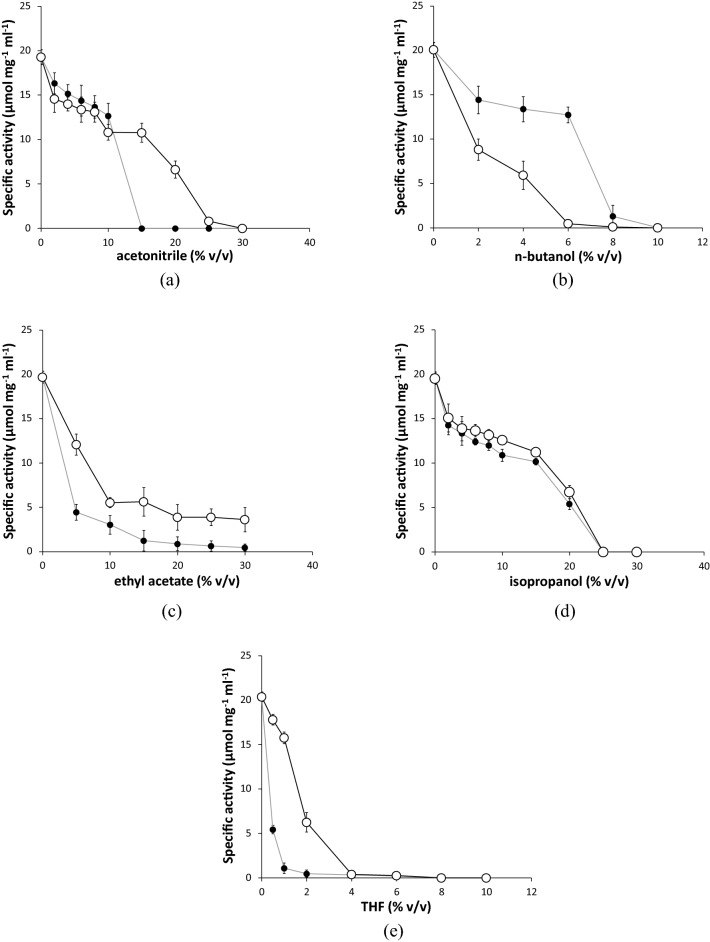
Retention of transketolase catalytic activity after pre-incubation with increasing concentrations of organic solvents. (open circle) Apo-TK and (filled circle) holo-TK were compared for their stability to incubation with (**a**) acetonitrile (**b**) n-butanol (**c**) ethyl acetate (**d**) isopropanol (**e**) THF, in 50 mM Tris–HCl, pH 7.0 (plus 4.8 mM TPP, 18 mM MgCl_2_ for holo-TK), for 3 h at 25 °C and 1000 rpm shaking. Enzyme activity was measured after a 20-fold dilution of the solvents, at 50 mM HPA and 50 mM GA in 2.4 mM TPP, 9 mM MgCl_2_, 50 mM Tris–HCl, pH 7.0.

**Figure 3:**
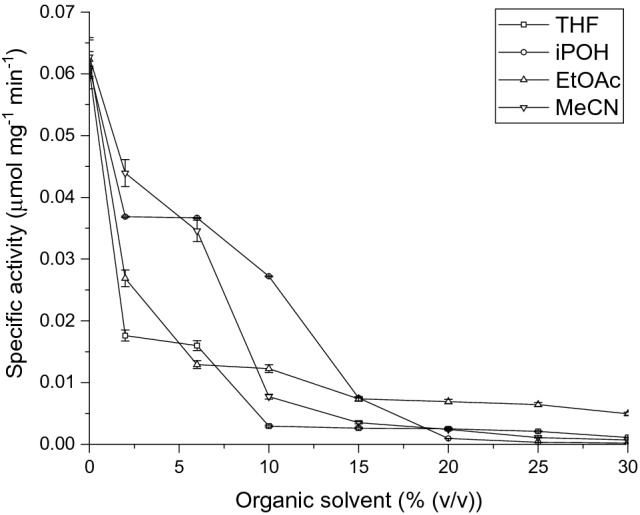
Retention of transketolase catalytic activity in cofactor-free systems with increasing concentrations of organic solvents. Apo-TK was compared for its stability to incubation with (open square)) THF, (open circle) isopropanol, (open triangle) ethyl acetate and (inverted open triangle) acetonitrile, in 50 mM Tris–HCl, pH 7.0 (plus 4.8 mM TPP, 18 mM MgCl_2_ for holo-TK), for 3 h at 25 °C and 1000 rpm shaking. Enzyme activity was measured after a 20-fold dilution of the solvents, at 50 mM HPA and 50 mM GA in 2.4 mM TPP, 9 mM MgCl_2_, 50 mM Tris–HCl, pH 7.0. Error bars represent one standard deviation about the mean (n = 3).

The results shown in Fig. 2 compare the effect of increasing the final solvent concentrations, upon the activity retained after 3 h, for apo-TK and holo-TK. As a control, we also measured the impact of the co-solvents on the residual activity of apo-TK in the absence of any added cofactors (Fig. 3) which is due to binding of endogenous cofactors during protein expression. This confirmed that the residual enzyme activity was 286-fold less than for the co-solvent-free holo-TK, as observed previously in lysates^64^. The activity from the residual holo-TK within the apo-TK preparation, also had a similar dependence on the co-solvent concentrations as the holo-TK preparations.

In nearly all cases, the increased concentration of co-solvents eventually reduced the remaining activity to zero (Fig. 2). Apo-TK was more stable than holo-TK to three of the solvents tested, but less stable than holo-TK in nBuOH, and essentially of the same stability as holo-TK in iso-propanol (iPrOH). For example, acetonitrile (AcCN) half-inactivated apo-TK and holo-TK at 20% (v/v) and 12% (v/v), respectively. Similarly, EtOAc half-inactivated holo-TK at approximately 3% (v/v), and at 6% (v/v) EtOAc for apo-TK. Nevertheless, apo-TK retained 18% residual activity at 30% (v/v) EtOAc, whereas holo-TK was already completely inactivated by 15% (v/v). The sharp decrease in activity up to 10% EtOAc and the apparent retention of activity above it for both apo and holo-TK potentially resulted from having reached the 8.3% (v/v) solubility of EtOAc in water^67^. Increasing the concentration at above 8.3% (v/v) EtOAc, formed emulsions which affected the deactivation profile of apo-TK and holo-TK. Holo-TK was completely inactivated by 15% (v/v), but not for apo-TK, even at 30% (v/v).

In iPrOH, both apo-TK and holo-TK behaved similarly, with a gradual drop-in activity until there was no activity in either case at 25% (v/v) iPrOH. Tetrahydrofuran (THF) was the most effective at deactivation, with holo-TK completely inactivated at just 2% (v/v) THF and apo-TK at 4% (v/v) THF. By contrast, apo-TK was less stable to nBuOH than holo-TK with concentrations of 2% (v/v) and 7% (v/v) respectively giving rise to 50% activity. For both apo-TK and holo-TK in nBuOH the residual activity tended to zero after reaching the 7.3% (v/v) solubility limit for nBuOH.

The generally lower tolerance of holo-TK to co-solvents compared to apo-TK is counterintuitive given that holo-TK is thermodynamically more stable than apo-TK. The dependence on solvent concentration is also clearly not simple. For example, AcCN, nBuOH and iPrOH displayed an initial lag at low solvent concentrations, where the activity decreased only slightly, before reaching a critical concentration at which deactivation occurred more abruptly. Such sigmoidal profiles can indicate a cooperative transition for structural unfolding or even the dissociation of protein dimers into monomers. By contrast, THF and EtOAc titrated out the activity rapidly with increasing concentration of co-solvent, suggesting either an isotherm for solvent binding to the protein, or that any cooperative structural transition was already well underway at the lowest concentration of co-solvent tested.

As apo-TK is already known to be less thermodynamically stable than holo-TK, and yet was found typically to be more co-solvent tolerant, TK inactivation by co-solvent did not appear to relate simply to the global conformational stability (or global denaturation) of the protein. Further biophysical characterisations were undertaken to elucidate whether inactivation was due to global protein denaturation, local protein unfolding, protein aggregation, or some other effect on the protein structure that is not directly detected, such as active-site binding. Before that we determined any relationships between the properties of the co-solvents and their potency for enzyme inactivation.

### Correlation of TK activity to calculated organic solvent properties

Although all the organic co-solvents in these experiments were polar, they showed considerable variability in their impact on retained TK activity, including their critical concentrations for inactivation, the sharpness of their deactivation curves, and also in their relative impacts upon apo-TK and holo-TK. The effectiveness of a polar solvent for inactivation of proteins might be expected to depend upon the polarity or hydrophobicity of the solvent, and also their ability to form hydrogen bonds in place of water.

**Figure 4:**
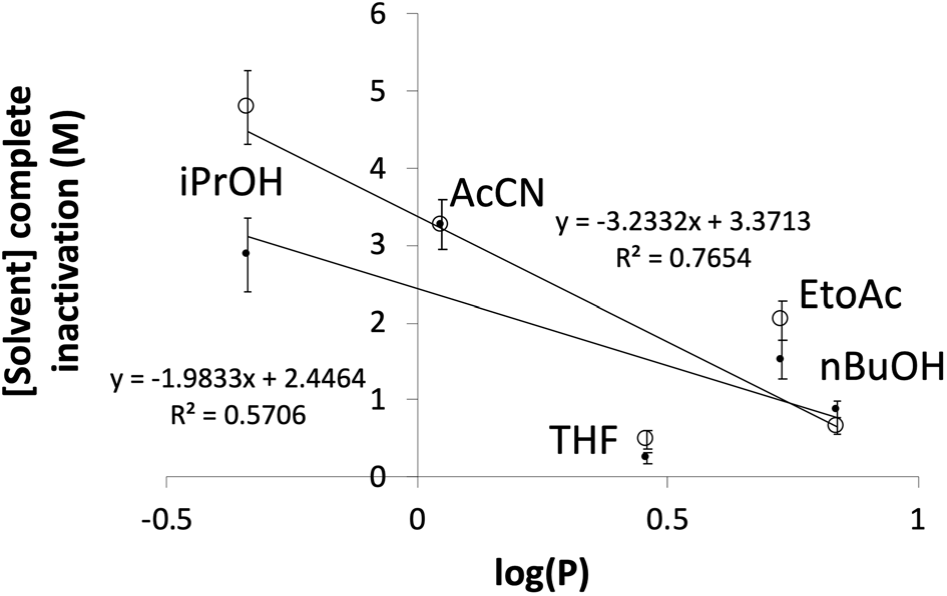
Correlations of solvent log(P) with their TK inactivation potency. Log(P) correlates well with the molar concentration required to inactivate either (open circle) holo-TK or (filled circle) apo-TK. Error bars represent one standard deviation about the mean (n = 3).

The polar co-solvents can be categorized as aprotic (AcCN, EtOAc and THF), or protic (nBuOH and iPrOH). This simple categorization had no obvious correlation to the concentrations of co-solvents required for complete enzyme inactivation. The characteristics of each co-solvent used in terms of various theoretically calculated and experimentally determined physicochemical properties, is shown in Table 1, along with the Pearson’s R^2^ values for linear correlations between each co-solvent property, and the molar solvent concentrations required for complete inactivation of holo-TK. Log(P) gave the best correlation for both apo-TK (R^2^ = 0.87) and holo-TK (R^2^ = 0.57) (Fig. 4). Other solvent properties correlated much less with the molar holo-TK inactivation concentration, such as the molecular weight (R^2^ = 0.4), molecular volume (R^2^ = 0.4), topological polar surface area (R^2^ = 0.3), number of potential hydrogen bonds (R^2^ = 0.1), dipole moment (R^2^ = 0.2), and the dielectric constant of the co-solvent (R^2^ = 0.4). Although the Log(P) correlation was poor, it showed the highest R^2^ which was consistent with previous observations that correlated hydrophobicity, and not the dielectric constant, to loss of enzyme activity and stability^25,68,69^. However, various mechanisms have been used to explain such previous correlations, and so we sought to further characterise the solvent-induced inactivation of TK, as summarised in Table 2 and described below.

**Table 1:** Correlation of holo-TK inactivation to physicochemical properties of the polar organic solvents.

Solvent	[S0]^a^ (% (v/v))	[S0]^a^ (M)	TPSA^b^ (Å^2^)	logP^c^	Vol^b^ (Å^3^)	MW^b^ (Da)	HB^d^	Dielectric constant^e^	Dipole^e^ (D)
THF	2	0.25	9.23	0.46	78	72	2	7.58	1.75
EtOAc	15	1.5	26.3	0.73	90.5	88	4	6.02	1.88
AcCN	15	2.9	23.8	− 0.34	46.1	41	1	37.5	3.44
iPrOH	25	3.3	20.2	0.05	70.6	60	2	19.9	1.66
nBuOH	8	0.9	20.2	0.84	87.6	74	2	17.5	1.75
R^2f^			0.3	0.57	0.4	0.4	0.1	0.4	0.2

^a^Concentration of solvent required for complete [S_0_] inactivation of holo-TK.

^b^Calculated using the Molinsky online software tool (http://www.molinspiration.com).

^c^Experimental values from Sangster^77^.

^d^Number of potential hydrogen bond acceptor and donor sites.

^e^Obtained from the Louisiana State University Macromolecular Studies Group Server (http://macro.lsu.edu/).

^f^R^2^ values are for linear Pearson correlations to [S_0_] in Molar.

**Table 2:** Effects of polar organic co-solvents on TK structure and activity after 3 h at 25 °C.

Solvent	[S]^a^ (%(v/v))	Activity at 3 h (%)		CD^b^ % native holo TK (± 2%)		CD initial rate unfolding^c^ (% h^−1^)		FLI transition^d^ (%(v/v))	DLS
		Holo	Apo	0 h	3 h	Holo	Apo	Holo	Holo
None	0	100	100	100	101	0	0	n/a	7 nm
THF	2	0	32	78	74	1.7	1.7	None	9 nm
EtOAc	10	15	31	99	98	1.2	1.4	None	8 nm
AcCN	20	0	35	90	54^f^	16.2	1.1	> 15%	> 1000 nm
iPrOH	20	30	35	100	95	1.4	1.0	> 22%	15 nm
nBuOH	8	10	0	98	98	0.8	2.1	7–8%	> 1000 nm

^a^Concentration of solvent added.

^b^CD % Native based on signal at 222 nm, except EtOAc which is based on signal at 223 nm to avoid a high dynode voltage at < 222 nm.

^c^Initial rate of unfolding was determined by converting mean residue ellipticity into % loss of native structure, and by subtracting the baseline rate determined in the absence of solvent.

^d^Fluorescence intensity.

^e^Time dependence of fluorescence intensity (approximate time taken to reach equilibrium).

^f^Aggregated after 4.5 h.

### Secondary structure of apo-TK and holo-TK in polar co-solvents

To determine the impact of co-solvents on the secondary structure content of TK, and also whether the enzyme was unfolding globally or partially, or aggregating, far-UV circular dichroism (CD) spectra were obtained at approx. 30–45 min intervals for between 3 and 22 h, for both apo-TK and holo-TK, using each volume fraction of solvent that resulted in ≈ 70% inactivation of the apo-TK enzyme (specific activity of 6 µmol mg^−1^ min^−1^), except for nBuOH which was increased to ensure holo-TK was also inactivated.

**Figure 5:**
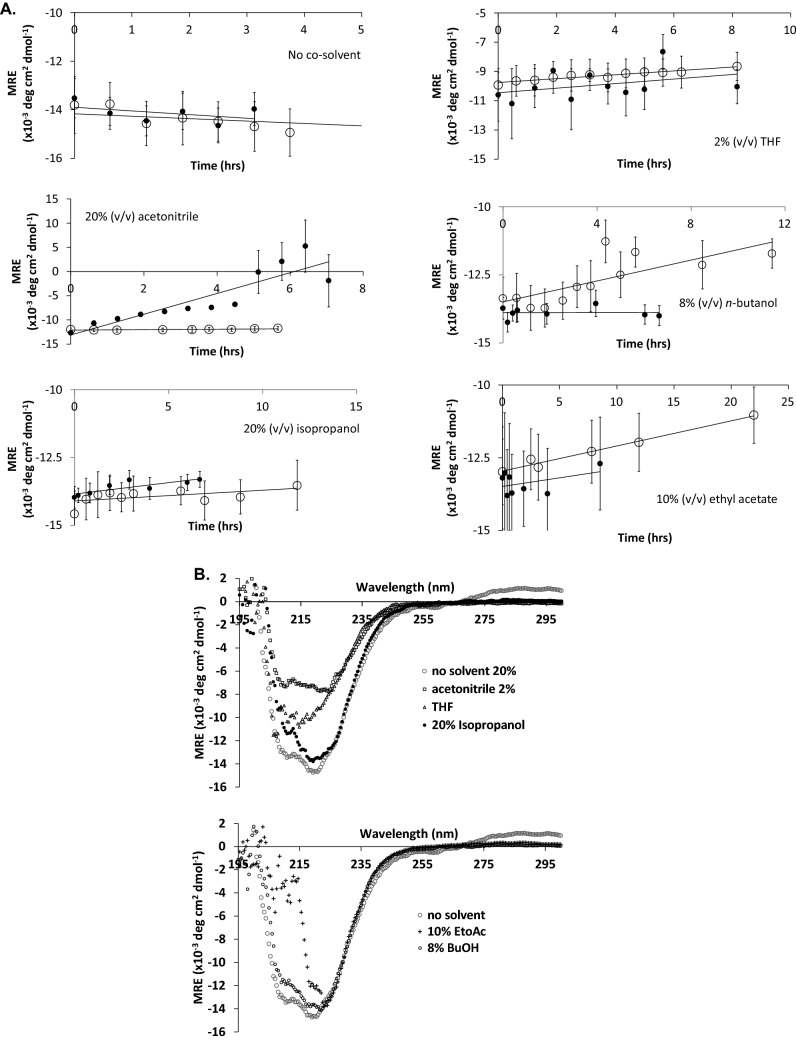
(**A**) Time-dependence of circular dichroism mean residue ellipticity at 222 nm, after mixing with co-solvents. (open circle) Apo-TK and (filled circle) holo-TK at 0.5 mg mL^−1^ in 25 mM Tris–HCl, pH 7.0, were incubated with no solvent, 20% (v/v) acetonitrile, 8% (v/v) n-butanol, 10% (v/v) ethyl acetate, 20% (v/v) isopropanol, or 2% (v/v) THF. Holo-TK also contained 2.5 mM MgCl_2_, 0.25 mM TPP. Error bars represent one standard deviation about the mean (n = 3). (**B**) Circular dichroism spectra of holo-TK after incubation with organic solvents. Holo-TK (0.5 mg mL^−1^) in 25 mM Tris–HCl, pH 7.0, 2.5 mM MgCl_2_, 0.25 mM TPP, and the presence of (open circle) no solvent; (open square) 20% acetonitrile; (open circle) 8% n-butanol (+) 10% ethyl acetate (filled circle) 20% isopropanol (open triangle) 2% THF, was incubated for 3 h at 25 °C before full spectra (195–300 nm) were acquired. Error bars represent one standard deviation about the mean (n = 3).

The time dependencies of the mean residue ellipticity at 222 nm for apo-TK and holo-TK in each co-solvent, and also without co-solvent, are shown in Fig. 5A, and the initial rates of secondary structure loss are shown in Table 2. The far-UV CD spectra of holo-TK, after incubation with co-solvents for 3 h, are also shown in Fig. 5B. It can be seen in Fig. 5A that the control samples of apo-TK and holo-TK with no added co-solvent, each showed no loss of secondary structure content over the course of at least 4 h. By contrast, relative to the control samples, the co-solvents each induced small initial losses of secondary structure at rates of between 1.0 and 2.1% h^−1^ for apo-TK, and between 0.8 and 1.7% h^−1^ for holo-TK, except for AcCN with holo-TK which led to a more rapid loss of 16% h^−1^. Apo-TK and holo-TK had similar rates in any given solvent, except for in AcCN where holo-TK lost secondary structure at a rate that was 15-fold faster than for apo-TK, and conversely for nBuOH where apo-TK had a 2.7-fold higher rate than holo-TK. AcCN with holo-TK was the only sample for which aggregates were detected by CD as indicated after 4.5 h of secondary structure loss, by a sudden and simultaneous increase in the dynode voltage and mean residue ellipticity.

It can be seen from Fig. 5A that all samples, except for THF, began with the same initial secondary structure content, with a mean residue ellipticity at 222 nm of − 13,500 ± 500 deg cm^2^ dmol^−1^. THF by contrast appeared to begin with only 78% of the native structure (− 10,590 deg cm^2^ dmol^−1^), yet this only decreased to 74% after 3 h. The far-UV spectrum taken after 3 h in THF (Fig. 5B), and at all earlier time-points (not shown), indicated that the smaller ellipticity at 222 nm was due to distortion away from the spectrum expected from a predominantly α-helical protein. This was due to absorbance flattening by THF, that gave a correspondingly increased dynode voltage at 224 nm and below (not shown), to above that acceptable for the instrument, rather than resulting from any actual loss of native structure or conformational change immediately after the addition of THF. EtOAc was similarly affected by a high dynode voltage at below 222 nm leading to considerable signal scattering at below 215 nm at all time-points.

**Figure 6:**
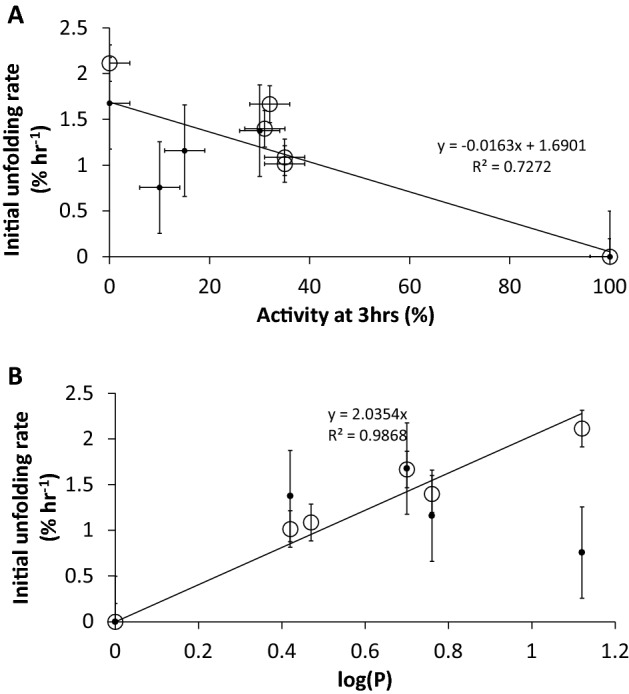
(**A**,**B**) Relationship between the local unfolding rate, retained activity after 3 h, and log(P). Both plots exclude MeCN as this solvent unfolded the protein globally. Apo-TK (open circle) and holo-TK (filled circle) Error bars represent one standard deviation about the mean (n = 3).

From Fig. 6A,B it can be seen that the initial rates of secondary structure loss determined by CD were found to correlate well (R^2^ of 0.91 for apo-TK, 0.71 for holo-TK, and 0.73 combined) to the activity retained after 3 h of incubation with the co-solvents, and also to the log(P) of the co-solvents (R^2^ of 0.94 for apo-TK), when excluding the case of AcCN for holo-TK. This indicated that the inactivation of the enzyme by co-solvent for both apo-TK and holo-TK was linked to the slow loss of secondary structure observed in most samples. Furthermore, as the secondary structure loss of only 0.8–2.1% h^−1^ led to 70–100% inactivation in apo-TK and holo-TK, then the far-UV CD signal change was most likely due to local destabilisation and unfolding, including at one or more functionally critical elements of structure within the entire protein population, rather than global unfolding of only 2.1% of the protein population.

For AcCN with holo-TK, the significantly higher rate of unfolding, led to 54% native structure after 3 h, and indicated that AcCN had induced global unfolding, from which aggregation then occurred after 4.5 h. Holo-TK was completely inactive after 3 h, indicating that global unfolding could only account for up to 54% of the inactivation mechanism, and therefore that the local unfolding observed with apo-TK in AcCN, had additionally inactivated holo-TK.

### Particle size distributions from dynamic light scattering

Dynamic light scattering (DLS) was used to measure particle size distributions of holo-TK in the presence of the co-solvents (see supplementary Fig. S1). Holo-TK in the absence of organic co-solvent gave a particle size of 7–8 nm as expected theoretically from the native homodimer structure^70^. At 20% (v/v) AcCN and 8% (v/v) nBuOH, holo-TK at 0.1 mg ml^−1^ contained DLS-detectable aggregates of 1000–4000 nm within 1 h of incubation. At 20% (v/v) iPrOH a very small aggregate peak at 400 nm was observed within 1 h, but more significantly, the monomer peak had shifted to approximately 15 nm indicating either the formation of a soluble oligomer, a shifted monomer peak due to viscosity effects, the formation of a solvent boundary layer, or otherwise significant protein unfolding. In 10% (v/v) EtOAc, only the native-like peak was detected at 8 nm indicating the retention of the homodimer, and no aggregation. For 2% (v/v) THF, the native-like peak increased slightly within 1 h to 9 nm, indicating a homodimer with the peak-shift due to altered solvent viscosity, although not inconsistent with partial local unfolding, or the formation of a significant solvent boundary layer associated with the homodimer. No aggregates were detected with THF.

It should be noted that by DLS, the % volume is proportional to the cube of the particle diameter, and hence even very small amounts of aggregates can suppress the detection of the native monomer peak. Therefore, it is very possible that the monomer, or even the 15 nm state observed for iPrOH, was present also for AcCN or nBuOH, but suppressed by the presence of larger (> 1000 nm) aggregates. In iPrOH, the smaller (400 nm) aggregate was clearly a very low-populated aggregate which allowed the 15 nm state to be observed. Overall, these results are consistent with the emergence of low levels of aggregates in nBuOH and iPrOH, more significant aggregation in AcCN as detected also by CD, and no aggregation observed by any method in EtOAc or THF. Therefore, aggregation was not well correlated to enzyme inactivation, as all solvents led to at least 70% inactivation of holo-TK. Only the significant levels of aggregate observed by CD could potentially contribute to enzyme inactivation for holo-TK, but this only occurred after 4.5 h of incubation, and for one solvent only (AcCN).

### Fluorescence spectroscopy of apo-TK and holo-TK in polar co-solvents

Fluorescence tryptophan spectroscopy has been previously successfully applied to provide tertiary structural information for TK^62,63,66,71^. For *E. coli* TK, there are 11 tryptophan residues per monomer which dominate the intrinsic fluorescence signal. These are distributed across the entire structure, with three fully buried and eight partially solvent exposed within each monomer, three located at or close to the dimer interface, and one of those being within one of the cofactor-binding loops. Thus, the intrinsic fluorescence of *E. coli* TK is responsive to global and local unfolding, dimer dissociation and aggregation, and can lead to both an increase or decrease in intrinsic fluorescence intensity, as observed previously where urea denaturation gave an initial increase followed by a decrease at higher urea concentrations^63^. By contrast, thermal unfolding results in immediate aggregate formation which gives an increase in the fluorescence intensity of TK as a single cooperative transition due to a net burial of the partially exposed tryptophan residues.

**Figure 7:**
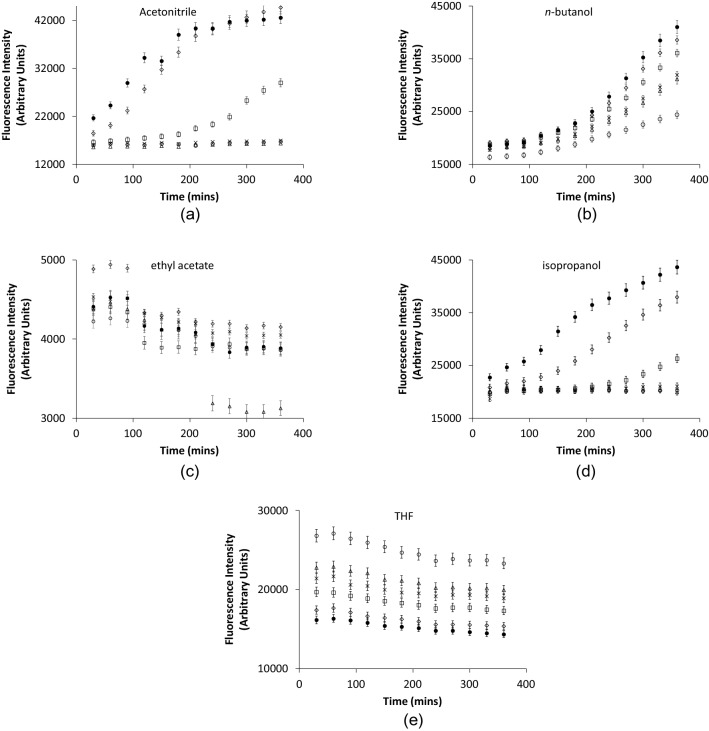
Time dependence of fluorescence intensity at various organic solvent concentrations for holo-TK. Fluorescence intensity was measured at 340 nm with excitation at 280 nm. Holo-TK at 0.1 mg mL^−1^, in 5 mM MgCl_2_, 0.5 mM TPP, 25 mM Tris–HCl, pH 7.0, was measured every 30 min for 6 h at 25 °C in (open circle) 5%, (open triangle) 10%, (x)15%, (open square) 20%, (open rhombus) 25% and (filled circle) 30% (**a**) acetonitrile (**b**) n-butanol (**c**) ethyl acetate (**d**) isopropanol (**e**) THF. Error bars represent one standard deviation about the mean (n = 3).

Figure 7 shows the time-dependence of intrinsic fluorescence intensities, for holo-TK in 5–30% (v/v) co-solvent. EtOAc and THF both resulted in steady decreases in fluorescence over time, for all concentrations tested. EtOAc at 10% (v/v) led to a 7% decrease in fluorescence, and THF at 5% (v/v) led to a 9% decrease, each after 3 h of incubation. These results are consistent with the local unfolding without aggregation as observed by CD above. By contrast, the fluorescence intensities for AcCN, nBuOH and iPrOH, resulted in lag-phases followed by rapid increases in fluorescence. The rapid increase occurred progressively earlier with increasing co-solvent concentration, and for AcCN at least the lag phase disappeared at the highest concentrations. This lag-phase behaviour is typical of aggregation kinetics^72^, and the fluorescence increases occurred in the same samples for which aggregation was observed by DLS. For 20% (v/v) AcCN, the fluorescence intensity increased over a timescale consistent with the global unfolding and aggregation observed by CD. For AcCN, nBuOH and iPrOH, it remained possible that the fluorescence changes indicative of aggregation, were also convoluted with contributions from the local unfolding events observed by CD.

**Figure 8:**
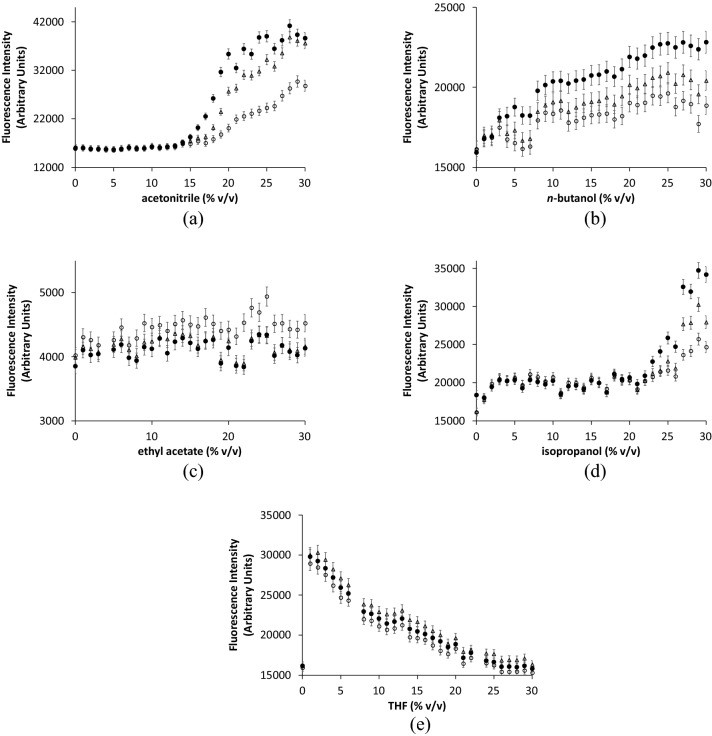
Fluorescence intensity measurements of apo-TK after incubation with increasing concentrations of organic solvents. Fluorescence intensity was measured at 340 nm with excitation at 280 nm. Apo-TK at 0.1 mg mL^−1^ in 25 mM Tris–HCl, pH 7.0 was incubated with (**a**) acetonitrile (**b**) n-butanol, (**c**) ethyl acetate (**d**) isopropanol (**e**) THF for (open circle) 1 h, (open triangle) 2 h, (filled circle) 3 h at 25 °C prior to measurements. Error bars represent one standard deviation about the mean (n = 3).

**Figure 9:**
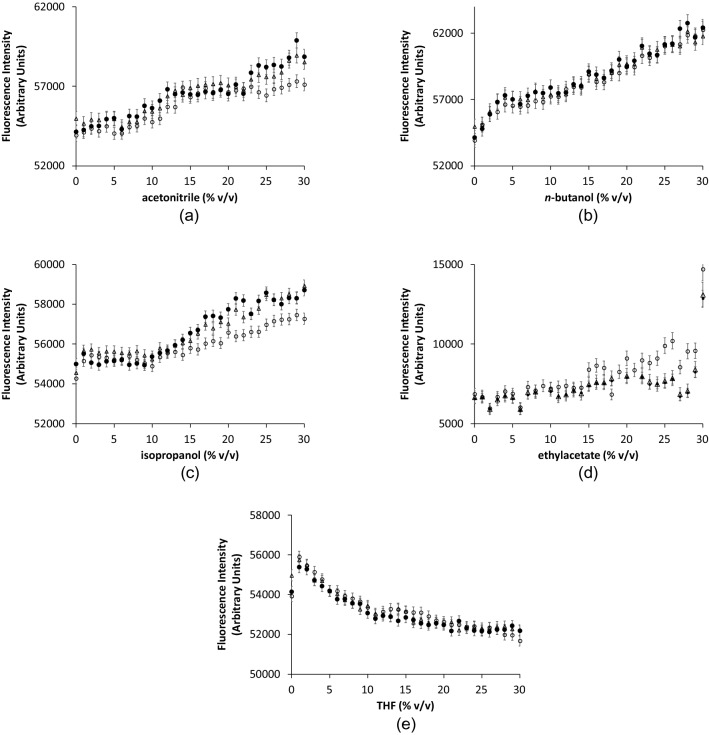
Fluorescence intensity measurements of holo-TK after incubation with increasing concentrations of organic solvents. Fluorescence intensity was measured at 340 nm with excitation at 280 nm. Holo-TK at 0.1 mg mL^−1^ in 5 mM MgCl_2_, 0.5 mM TPP, 25 mM Tris–HCl, pH 7.0, was incubated with (**a**) acetonitrile (**b**) n-butanol (**c**) ethyl acetate (**d**) Isopropanol (**e**) THF for (open circle) 1 h, (open triangle) 2 h, (filled circle) 3 h at 25 °C prior to measurements. Error bars represent one standard deviation about the mean (n = 3).

The effect of increasing co-solvent concentration on intrinsic fluorescence, as measured after 1, 2, and 3 h of incubation, is shown for apo-TK in Fig. 8 and holo-TK in Fig. 9. For apo-TK, the addition of co-solvents resulted in up to a 10% increase in the intrinsic fluorescence after 3 h in AcCN, nBuOH, iPrOH and EtOAc (Fig. 7), consistent with local rather than global unfolding. Increases were monotonic for 0–30% (v/v) AcCN, 4–30% (v/v) nBuOH, 10–30% (v/v) iPrOH, and 0–29% (v/v) for EtOAc. Below 10% (v/v) iPrOH there was no change, and at 30% (v/v) EtOAc there was a small increase. For THF, the fluorescence intensity decreased exponentially, with most of the change completed over 0–10% (v/v). The lack of obvious transitions for AcCN, EtOAc and THF, and yet clear transitions at 0–4% (v/v) nBuOH, and 10% (v/v) iPrOH, were consistent with the inactivation profiles for apo-TK in Fig. 3.

For holo-TK the addition of co-solvents resulted in similar profiles to those for apo-TK, in EtOAc and THF (Fig. 9). AcCN and iPrOH each gave no change initially and then increased sharply at above 15% (v/v) and 22% (v/v) respectively. In nBuOH, the fluorescence intensity showed a small increase that was monotonic overall, but with a small and sharp increase at 7–8%. These three sharp transitions are larger than any transitions observed in apo-TK, which didn't aggregate, and they are consistent with the aggregation behaviour of holo-TK observed as time-dependent lag-phases in Fig. 7, and by DLS. Again, the lack of obvious transitions for EtOAc and THF, and yet clear transitions at 15% (v/v) AcCN, 7–8% (v/v) nBuOH, and 22% (v/v) iPrOH, are consistent with the inactivation profiles for holo-TK in Fig. 3. However, the aggregate formation for holo-TK tended to occur at slightly higher solvent concentrations than the holo-TK inactivation, suggesting that it occurred after inactivation, and therefore that aggregates formed from an already inactive enzyme. This was consistent with the earlier observation by CD that inactivation was primarily due to a local unfolding event (or global unfolding in the case of AcCN with holo-TK), and that in some cases for holo-TK this also led to aggregation.

## Discussion

The deactivation of both apo-TK and holo-TK by co-solvents generally resulted from a local unfolding event that retained a near-native homodimeric structure. The only exception was for holo-TK in 20% (v/v) AcCN, which induced global unfolding. This is consistent with previous studies that suggest enzyme activity loss in polar solvents is primarily due to the stripping of water from the protein surface and enzyme active-site, which in turn affects the active-site geometry, stability and catalytic function^73,74^. Local unfolding also led to aggregation for holo-TK in certain solvents, but only after enzyme deactivation had already occurred. For holo-TK in AcCN, enzyme inactivation was dominated by global unfolding.

The most hydrophobic co-solvents, with higher log(P) values, deactivated the enzyme at proportionally lower molar concentrations of co-solvent, although THF appeared to inactivate at lower concentrations than expected from this trend. The log(P) values also correlated well to the local unfolding rate observed by CD, indicating that the more hydrophobic solvents were better able to unfold the local structure responsible for enzyme deactivation.

Previous studies of other enzymes found that alcohols tend to disrupt tertiary structure and leave the secondary structure interactions largely undisturbed^75^. The data for holo-TK were not entirely consistent with this conclusion, as local changes in secondary structure correlated with activity loss. By contrast, the intrinsic fluorescence indicated little tertiary structure unfolding, whereas the increases in intrinsic fluorescence intensity, and the lag-phases in their kinetics observed for holo-TK in iPrOH or nBuOH, were most likely due to aggregate formation. However, from CD and DLS measurements, any low levels of aggregate formation were still insufficient to explain the activity loss for TK. More recently, the prolonged exposure to organic solvents was proposed to lead to a decrease in enzyme dynamics and active-site polarity, and also the reorientation of active site residues, which in turn affect the ionization state of the catalytic residues, and hence the stability of transition states and intermediates required for catalysis^76^. However, these mechanisms would be rapidly-reversible effects and so do not readily explain the loss of TK activity which was largely irreversible or at least very slow on the timescale of the activity assays which required a 20-fold dilution out of the aqueous solvent mixture.

Interestingly, for THF, AcCN, and EtOAc, the more thermodynamically stable holo-TK did not retain more activity than the apo-TK after exposure to these co-solvents, indicating that solvent tolerance was not simply correlated to global conformational stability. However, the local unfolding rates were similar between apo-TK and holo-TK, and so it appears that the local unfolding measured by CD was also not due to unfolding of the cofactor loops, as these are already unstructured in apo-TK. However, the structure of the cofactor loops, and/or presence of bound cofactors adversely influenced the retention of activity. Apo-TK unfolding was found previously to be more reversible than for holo-TK, when refolding TK from urea^63^. If the unfolding of local structure within or close to the active site was more reversible in apo-TK than in holo-TK, then that might explain why the 20-fold dilution into the reaction assay recovered more activity for apo-TK than for holo-TK.

It was also interesting that only AcCN induced global unfolding, and CD-observable aggregation, and then only in the holo-TK form. AcCN had one of the lowest log (P) values of the co-solvents tested, and so simple hydrophobicity of the solvent was not the reason. AcCN may be able to form additional interactions with the protein, not found for the other co-solvents, that enable it to unfold the protein more effectively. Alternatively, the apparent unfolding observed by CD may actually also have been due to aggregation, in which case the properties of AcCN promote more irreversible unfolding and aggregation than for the other solvents.

The ideal conditions for biocatalysis in industry require that the enzyme remains active for the duration of the biotransformation. Therefore, even the slow unfolding or aggregation by solvents, as observed for iPrOH, nBuOH and AcCN is potentially problematic for longer reactions, or for repeated enzyme use. Considering this, TK was most tolerant in 15% (v/v) (2.0 M) iPrOH, 10% (v/v) (1.9 M) AcCN, or 6% (v/v) (0.65 M) nBuOH as in these cases, the activity remained at > 75%, whereas no aggregation was observed over 3 h.

Finally, this work has provided useful insights that will guide the future engineering of TK and other similarly complex enzymes. Apo-TK and holo-TK differ specifically in the organisation of their cofactor-binding loops. Given their different behaviours in the presence of co-solvents, improvements might be obtained by focusing enzyme engineering within the cofactor-binding loops and neighbouring regions close to the protein surface. A similar strategy was effective previously for the improvement of TK thermostability^48,65^.

## Methods

All chemicals and solvents were obtained from Sigma-Aldrich (Gillingham, UK) unless noted otherwise.

### Expression and purification of transketolase

N-terminally His6-tagged wild-type E. coli transketolase was expressed from *E. coli* XL10-Gold (Stratagene, La Jolla, CA) containing the engineered plasmid pQR791, purified as described previously^63^, dialysed at 4 °C for 24 h against 25 mM Tris–HCl, pH 7.0, and stored at 4 °C for a maximum of two weeks without loss of activity, and with no precipitation visible. Protein concentration was determined by absorbance at 280 nm, assuming a monomeric molecular weight (MW) of 72,260.82 g mol^−1^ and an extinction coefficient (e) of 93,905 L mol 1 cm^−1^^63^. The protein obtained through this overexpression is known to be essentially apo-TK with very low residual activity due to endogenous cofactors^64^.

### Residual activities after incubation with organic solvents

Samples were divided into two groups. The first determined the impact of solvents on holo-TK. 40 μL of 2.6 mg mL^−1^ apo-TK (in 125 mM Tris–HCl, pH 7.0) was mixed with 5 µL of a 20× cofactor stock (48 mM TPP, 180 mM MgCl_2_). This was incubated for 20 min at 25 °C with 1000 rpm shaking, before adding 55 µL of a range of solvents at different concentrations in water to yield 1 mg mL^−1^ holo-TK solutions (2.4 mM TPP, 9 mM MgCl_2_) in 50 mM Tris–HCl, pH 7.0. These solutions were then incubated for 3 h at 25 °C 1000 rpm. For the second group, apo-TK solutions received the same treatment as the described above for holo-TK, except that 5 µL of water were added instead of the 5 µL of the 20× cofactor stock solution. The activity of the apo-TK was subsequently measured after co-solvent incubation, both in the presence and absence of co-factors, to confirm the low residual activity of the apo-TK.

All residual activities were determined by addition of 15 µL enzyme solution samples to 285 µL of substrate stock. Substrate stock was in 50 mM Tris–HCl, pH 7.0 for holo-TK experiments and cofactor-free apo-TK experiments, but in 1.05× cofactors and 50 mM Tris–HCl, pH 7.0 for apo-TK experiments. These gave final concentrations of 50 mM HPA, 50 mM glycolaldehyde, 2.4 mM TPP (0 mM for cofactor-free apo-TK), and 9 mM MgCl_2_ (0 mM for cofactor-free apo-TK), in 50 mM Tris–HCl, pH 7.0, and 20-fold dilution of the original solvent. At regular intervals during a period of 90 min, aliquots of 20 µL were diluted 1:10 with 180 µL 0.1% (v/v) TFA, to stop the reactions and then transferred into 96 micro-well plates for measuring of products by HPLC as described previously^49^. Samples were loaded on a 300 mm Aminex HPX-87H column (Bio-Rad Laboratories) maintained at 60 °C, and analysed with an isocratic flow of 0.1% (v/v) TFA in water at 0.6 mL min^−1^.

### Secondary structure monitored by circular dichroism (CD)

CD spectra (190–300 nm) were recorded on an AVIV 202 SF spectrometer (AVIV Associates, Lakewood, NJ) at 25 °C using a 1 mm path length quartz precision cell cuvette. Samples were prepared as above at 0.5 mg mL^−1^ transketolase both with and without 2.5 mM MgCl_2_ and 0.25 mM TPP for holo-TK and apo-TK respectively, in 25 mM Tris–HCl, pH 7.0, and with the required % (v/v) solvent. The lower cofactor concentrations are sufficient for > 90% saturation of the TK (Miller, 2007), while minimising the dynode voltage in CD spectra due to absorbance by the cofactors. CD spectra were recorded at 0.5 nm intervals and averaged for 4 s at each wavelength. Spectra were recorded at 30–45 min intervals for up to 15 h after the addition of solvents. Spectra for 25 mM Tris–HCl, pH 7.0 buffer in the respective % (v/v) solvent was subtracted from each recording. Initial rates of structure change were obtained from linear fits to the plots of the CD signal at 222 nm over time incubated at 25 °C.

### Intrinsic fluorescence intensities

Holo- and apo-TK samples were prepared as above at 0.1 mg mL^−1^ either with or without 5 mM MgCl_2_ and 0.5 mM TPP, in 25 mM Tris–HCl, pH 7.0, and with the required % (v/v) solvent. Samples were incubated at 25 °C in UV transparent 96-well microplates (Costar, Corning Incorporated, NY, USA). Fluorescence intensity was measured every 30 min for 6 h from below the plate at 340 nm emission and 280 nm excitation using a FLUOstar microplate reader (BMG Labtechnologies Ltd., Aylesbury, UK). Different gain settings were used for the apo-TK and holo-TK experiments. Due to the interference with measurements by an incompatibility of EtOAc with the sample plates that appears after long incubations, those incubations were carried out in glass vials before transferring samples to UV transparent 96-well microplates immediately prior to measurements.

### Dynamic light scattering (DLS)

The particle size distributions of transketolase in the presence and absence of solvent was measured at 25 °C with a Zetasizer Nano S (Malvern Instruments Ltd., UK). Holo-TK at 0.1 mg mL^−1^ was prepared as above in 25 mM Tris–HCl, pH 7.0, with 0.5 mM TPP and 5 mM MgCl_2_ and the required % (v/v) solvent. Samples were incubated for 1, 2 and 3 h prior to data acquisition. Data were acquired in triplicate, with a 1 cm path length low volume disposable sizing cuvette. Control samples of the respective buffered solvents, with or without cofactors was subtracted from each recording. The hydrodynamic diameters of each sample were calculated from the averaged measurements using the Zetasizer Nano Series software V.4.20 (Malvern Instruments Ltd., Worcestershire, UK).

## Supplementary Information

Supplementary Figure S1.
