# Supplementary material for: Biophysical characterization of the inactivation of E. coli transketolase by aqueous co-solvents
Source: Sci Rep. 2021 Dec 8;11:23584. doi: 10.1038/s41598-021-03001-8 (PMC8654844; doi:10.1038/s41598-021-03001-8)
Supplement: Supplementary file 1 — Supplementary Figure S1. [file 41598_2021_3001_MOESM1_ESM.pdf]

Co-solvent-induced enzyme inactivation via local unfolding that is faster in the holo-enzyme.

Appendix A. Supplementary data

Figure S1 (a-c)

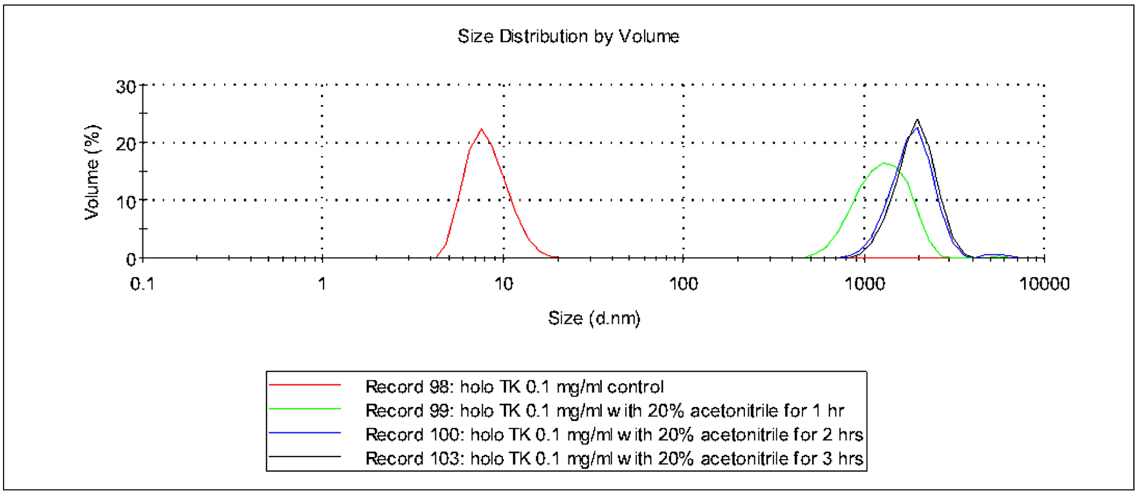

(a)

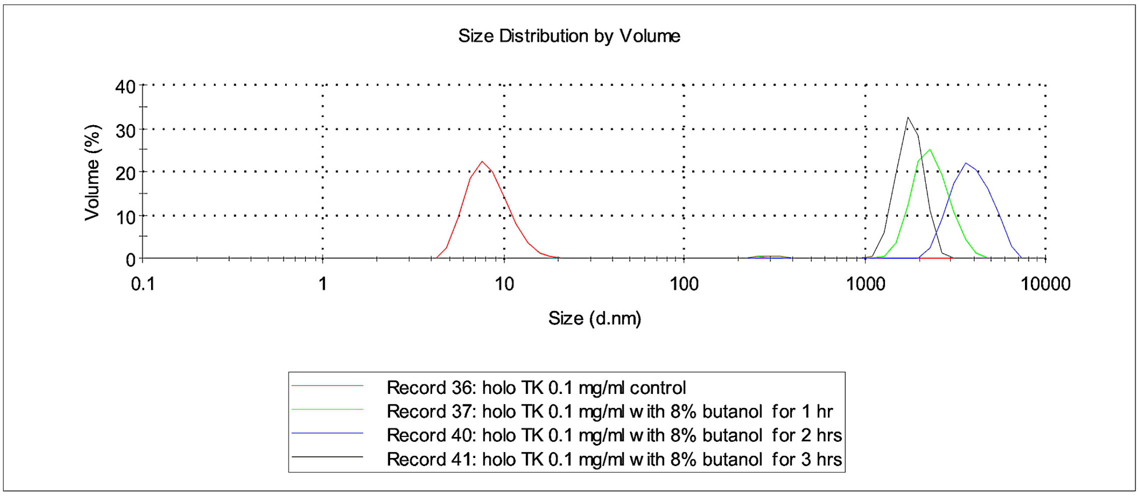

(b)

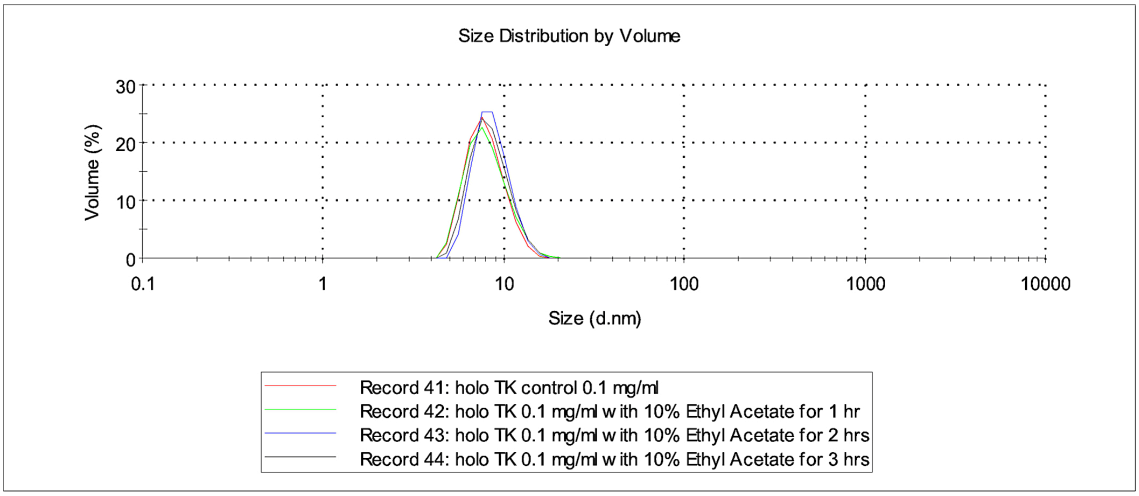

(c)

Figure S1 (d-e)

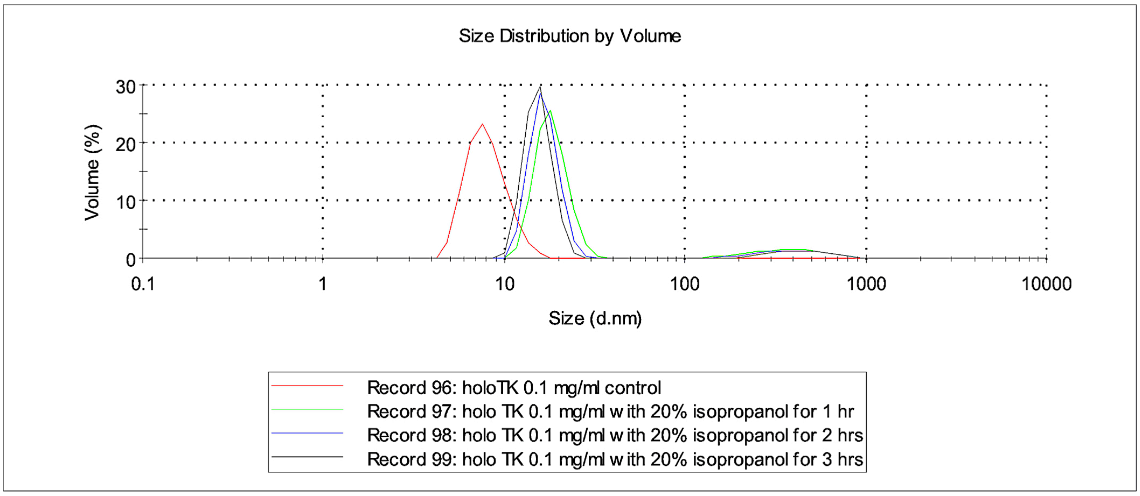

(d)

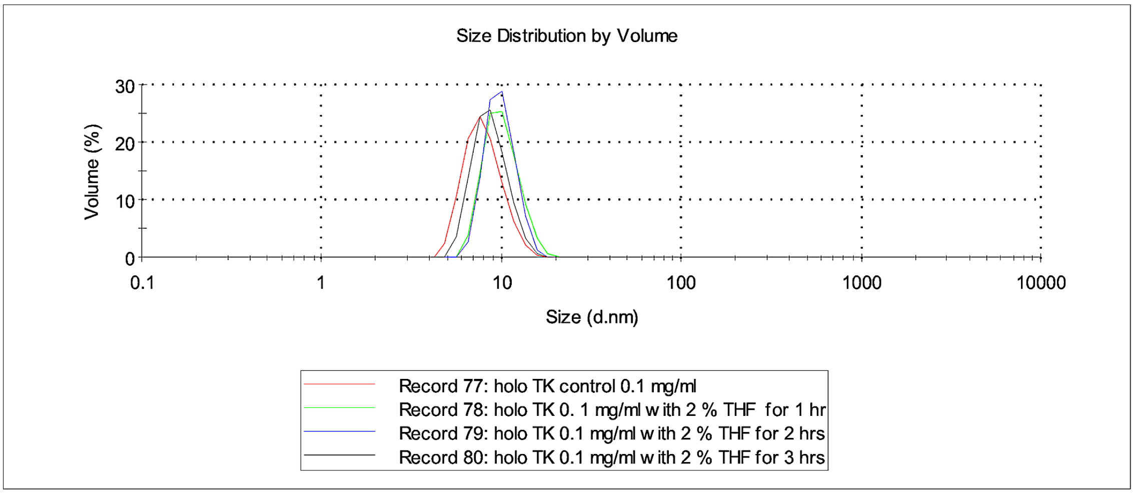

(e)

Figure S1. Size distribution volume (%) of holo-TK estimated by dynamic light scattering (DLS) in the presence of organic co-solvents. Samples contained 0.1 mg mL^{-1} protein in 25 mM Tris-HCl, pH 7.0, with cofactors (0.5 mM TPP and 5 mM $MgCl_2$), and (a) 20% acetonitrile, (b) 8% *n*-butanol, (c) 10% ethyl acetate, (d) 20% isopropanol or (e) 2% THF. Samples were incubated for 1, 2 and 3 h at 25 °C before measurement. Data from one replicate is shown for each sample type. Measurements were confirmed for triplicate samples.
